# Supplementary material for: Strigolactone perception and deactivation by a hydrolase receptor DWARF14
Source: Nat Commun. 2019 Jan 14;10:191. doi: 10.1038/s41467-018-08124-7 (PMC6331613; doi:10.1038/s41467-018-08124-7)
Supplement: Supplementary file 1 — Supplementary Information [file 41467_2018_8124_MOESM1_ESM.pdf]

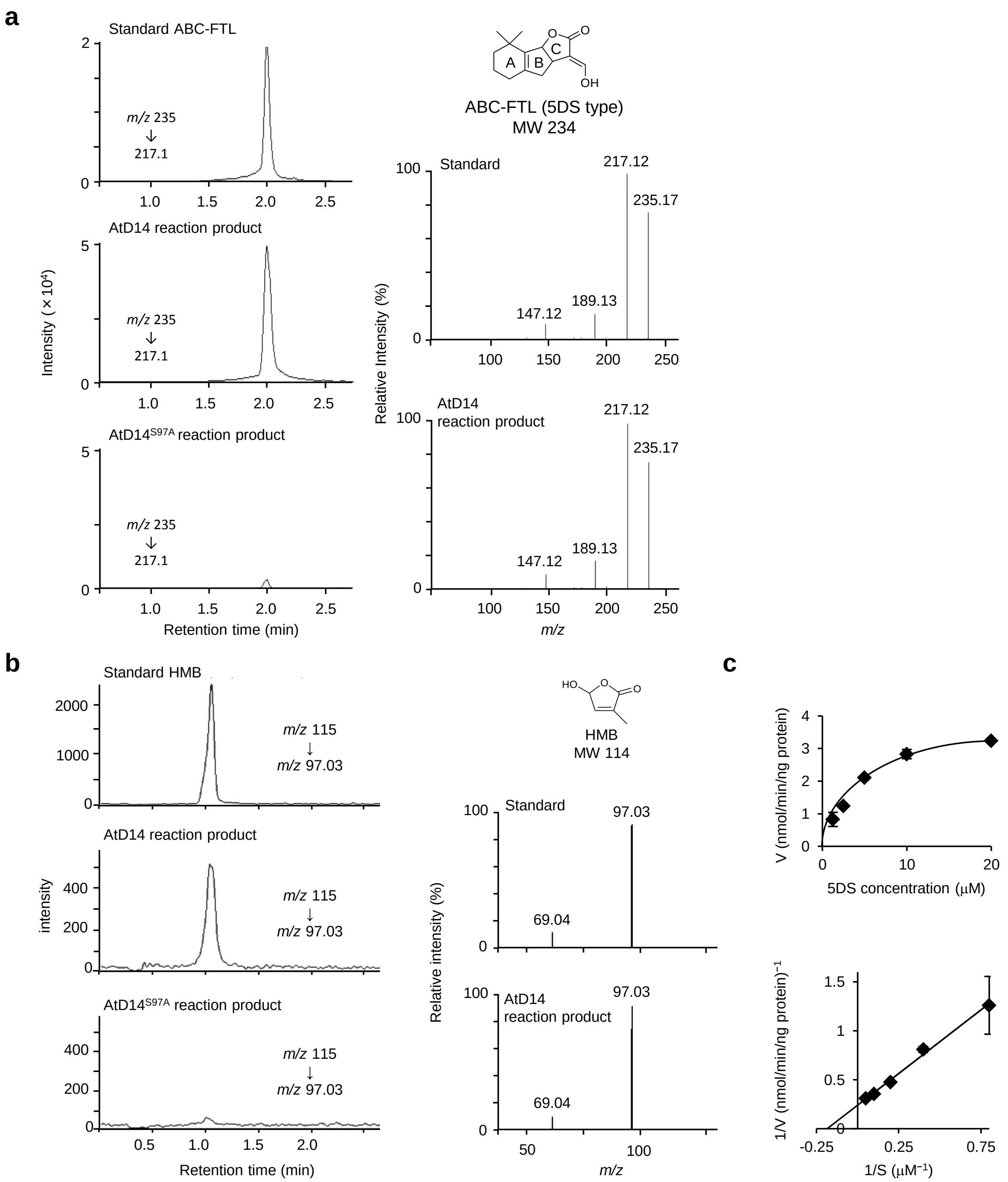

Supplementary Fig 1. Hydrolysis reaction analysis of AtD14 using 5DS as a substrate. a, LC-MS/MS analysis of ABC-FTL by a positive ion mode. Selected reaction monitoring (left) and full-scan spectra of fragment ions (right) of the standard ABC-FTL and a reaction product are shown. **b**, LC-MS/MS analysis of HMB by a positive ion mode. Selected reaction monitoring (left) and full-scan spectra of fragment ions (right) of standard HMB and a reaction product are shown. AtD14^{S97A}: AtD14 mutant protein whose catalytic triad Ser is replaced by Ala. **c**, The steady state kinetics study of AtD14 using 5DS as a substrate as was calculated by the amount of ABC-FTL. Upper panel; Hydrolase reactions of AtD14 with various concentrations of 5DS. Lower panel; Double reciprocal plots of reactions for 5DS by AtD14. The effect of substrate concentration on the reaction velocity was examined at various concentrations of 5DS. Data are the means ±SD (n=3). Source data are provided as a Source Data file.

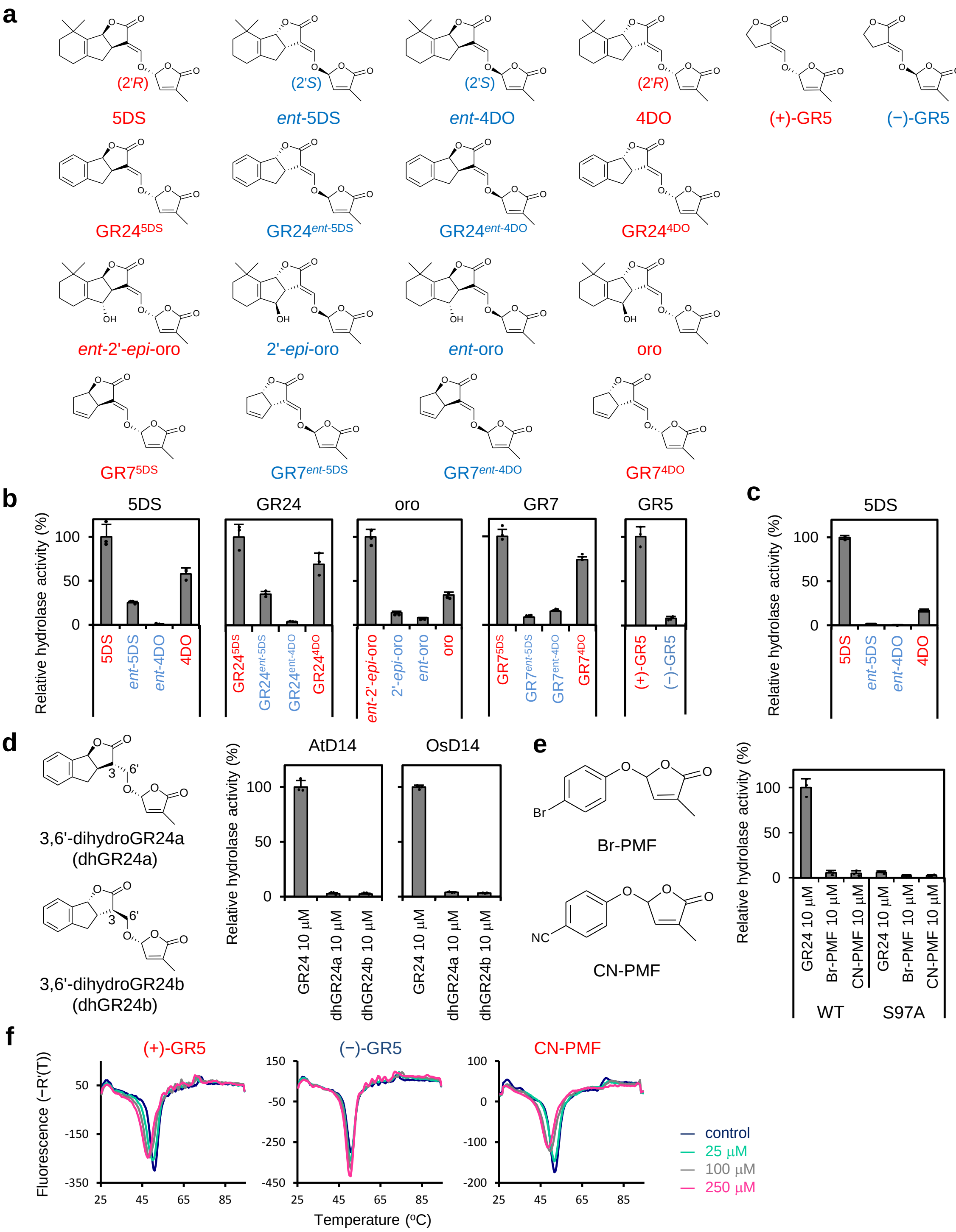

Supplementary Fig. 2. Biochemical function analysis of D14 using hydrolysis assays and DSF experiments. a, Chemical structures of each stereoisomer of SLs using in this study. The names in red and blue denote (2'*R*)- and (2'*S*)-isomers, respectively. **b**, Hydrolysis activity of AtD14 for stereoisomers of various SLs. 5DS, GR24, orobanchol (oro), and GR7 were incubated at 1 μM. GR5 was incubated at 10 μM. Data are the means ±SD (n=3). **c**, Hydrolysis activity of OsD14 for stereoisomers of 5DS at 1 μM. Data are the means ±SD (n=3). **d**, Relative hydrolase activity of AtD14/OsD14 with 3,6'-dihydroGR24. Among some stereoisomers of 3,6'-dihydroGR24, (2'*R*)-isomers were used as those structures were shown. Data are the means ±SD (n=3). **e**, Relative hydrolase activity of AtD14 with debranones. Data are the means ±SD (n=3). **f**, Melting temperature curves of OsD14 in the presencae of GR5 two isomers and a debranone, CN-PMF. The names in red and blue denote biologically active and inactive (or weakly active) compounds, respectively. Source data are provided as a Source Data file

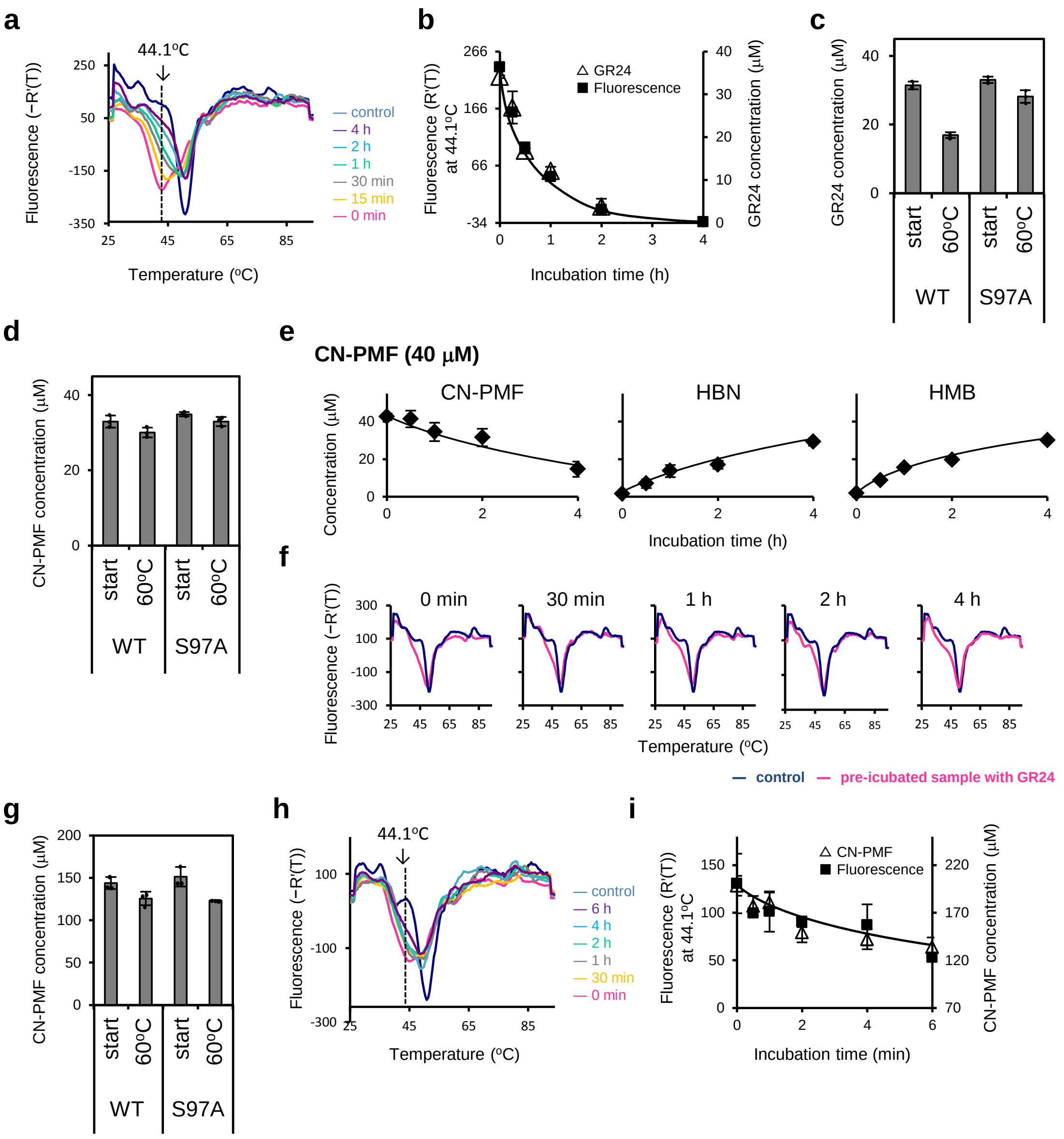

Supplementary Fig. 3. Time-course DSF experiments of AtD14 with GR24 or CN-PMF. a, Superimposed melting temperature curves of AtD14 pre-incubated with GR24 (40 µM) for indicated time period. Data are the means ±SD (n=3). **b**, The time-course plots of GR24 consumption (△) and the fluorescent intensity during the DSF operation at 44.1^{o}C (■), Tm of SL-bound form AtD14. Data are the means ±SD (n=3). **c**, Hydrolysis activity of AtD14 for GR24 (40 µM) during DSF operation. The DSF assays were stopped when the temperature reached to 60°C, and the remaining substrate was quantified using LC-MS/MS. Data are the means ±SD (n=3). **d**, Hydrolysis activity of AtD14 for CN-PMF (40 µM) during DSF operation. The DSF assay was stopped when the temperature reached to 60°C, and the remaining substrate was quantified using LC-MS/MS. Data are the means ±SD (n=3). **e**, Monitoring by LC-MS/MS of the AtD14 hydrolysis reaction of CN-PMF at 40 µM. Data are the means ±SD (n=3). **f**, Melting temperature curves of AtD14 pre-incubated with CN-PMF at 40 µM for indicated time period. **g**, Hydrolysis activity of AtD14 for CN-PMF (200 µM) during DSF operation. The DSF assay was stopped when the temperature reached to 60°C, and the remaining substrate was quantified by LC-MS/MS. Data are the means ±SD (n=3). **h**, Superimposed melting temperature curves of AtD14 pre-incubated with CN-PMF (200 µM) for indicated time period. **i**, The time-course plots of CN-PMF consumption (△) and the fluorescent intensity during the DSF operation at 44.1^{o}C (■), Tm of SL-bound form AtD14. Data are the means ±SD (n=3). Source data are provided as a Source Data file.

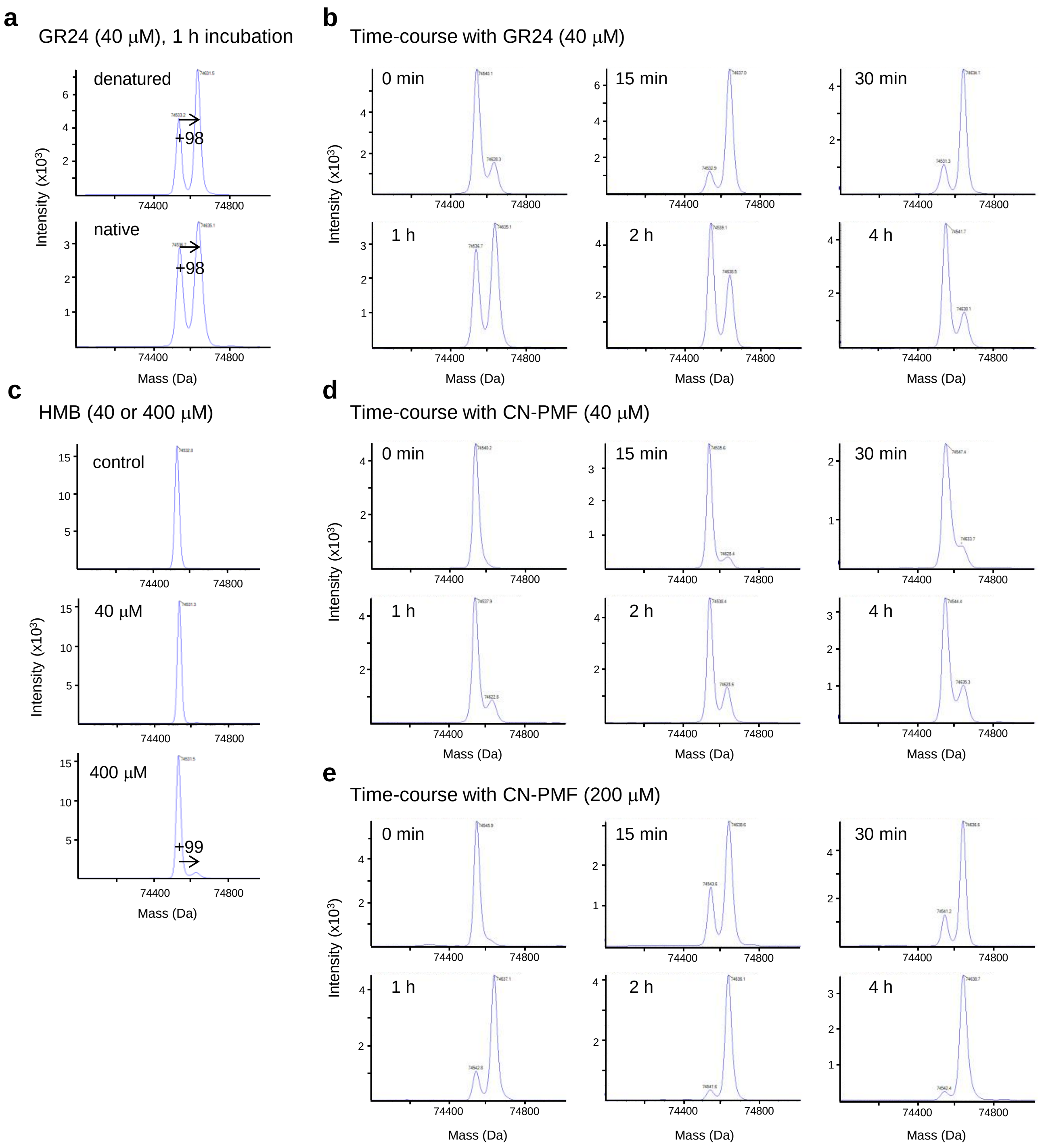

Supplementary Fig. 4. **Time-course analysis of the covalently-linked AtD14 protein using LC-MS/MS.** (**a)** Deconvoluted electrospray mass spectra of denatured (upper) or native (lower) MBP-AtD14 incubated with 40 μM GR24. (**b**) Time-course analysis of the modified AtD14 (native) during the hydrolysis reaction with 40 μM GR24. Deconvoluted electrospray mass spectra of MBP-AtD14 at each time point were shown. (**c**) Deconvoluted electrospray mass spectra of MBP-AtD14 (native) incubated with 40 or 400 μM HMB. (**d**) Time-course analysis of the modified AtD14 (native) during the hydrolysis reaction with 40 μM CN-PMF. Deconvoluted electrospray mass spectra of MBP-AtD14 at each time point were shown. (**e)** Time-course analysis of the modified AtD14 (native) during the hydrolysis reaction with 200 μM CN-PMF. Deconvoluted electrospray mass spectra of MBP-AtD14 at each time point were shown. The arrows in (**a**) and (**c**) indicates the mass increment of the adduct peak. Source data are provided as a Source Data file.

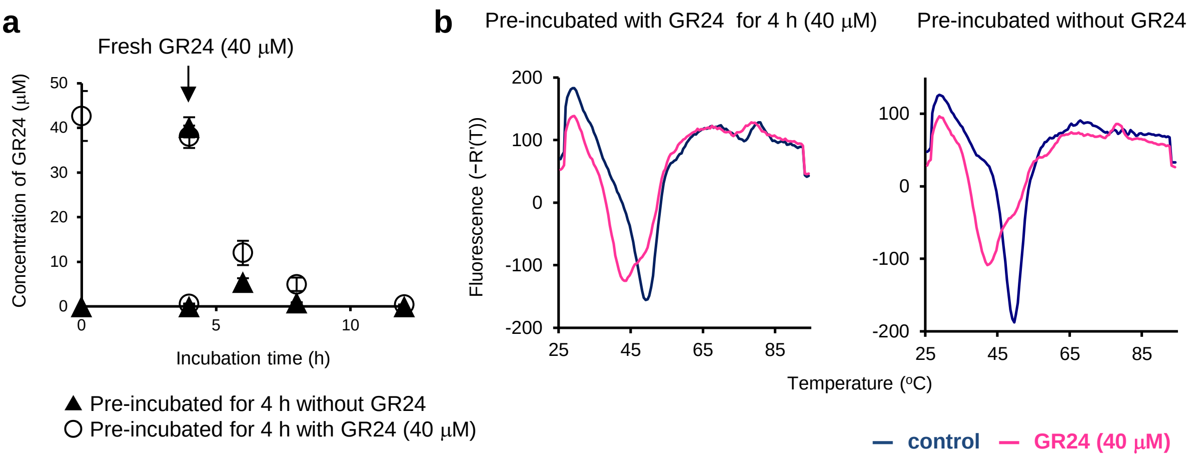

Supplementary Fig. 5., **Hydrolysis and DSF assays for freshly added GR24 using pre-incubated AtD14 with or without GR24. a**, Monitoring of the AtD14 hydrolysis reaction of GR24 using pre-incubated AtD14 with (○) or without (▲) GR24. Fresh GR24 at 40 μM final concentration was added at 4 h time period to the both samples. Data are the means ±SD (n=3). b. Melting temperature curves of AtD14 pre-incubated with or without GR24. Source data are provided as a Source Data file.

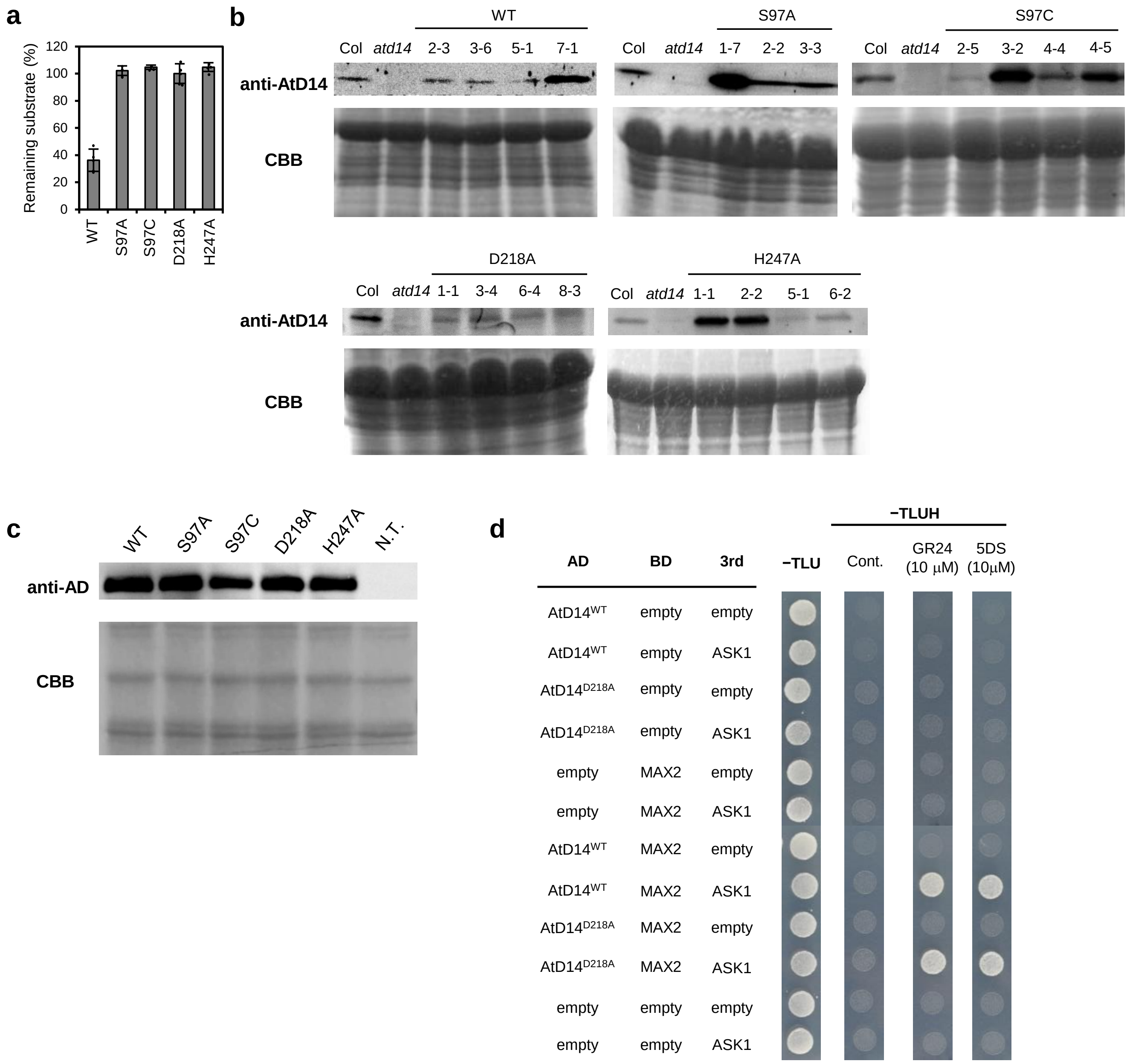

Supplementary Fig. 6. Functional analysis of catalytic triad mutants of AtD14. a, Hydrolase activity of AtD14 catalytic triad mutants using 1 μM of 5DS as a substrate. The remaining substrate, 5DS, was measured after hydrolysis reaction. Data are the means ±SD (n=3-5). **b**, Western blot analysis of AtD14 protein expression levels in transgenic plants expressing each AtD14 catalytic triad mutant in the Arabidopsis *atd14-2* mutant background as was detected by the AtD14 antibody. **c**, Western blotting analysis of AtD14 protein levels in the yeast transformant expressing each AtD14 catalytic triad mutant as was detected by the anti-AD antibody. N.T. indicates the non-transformed cells. Lower panel is the CBB stained picture. **d**, Y3H analysis of the interaction between MAX2 and a catalytic triad mutant, $AtD14^{D218A}$ in the presence of the 3rd protein, ASK1. Yeast transformants were spotted onto the control medium (SD −Leu/ −Trp/ −Ura (−TLU)) and selective medium (SD −Leu/ −Trp/ −Ura/ −His (−TLH)) in the absence or presence of SLs (10 μM GR24, or 10 μM 5DS). Control (Cont.) is acetone only. Source data are provided as a Source Data file. Uncropped blots can be found in the Source Data file.

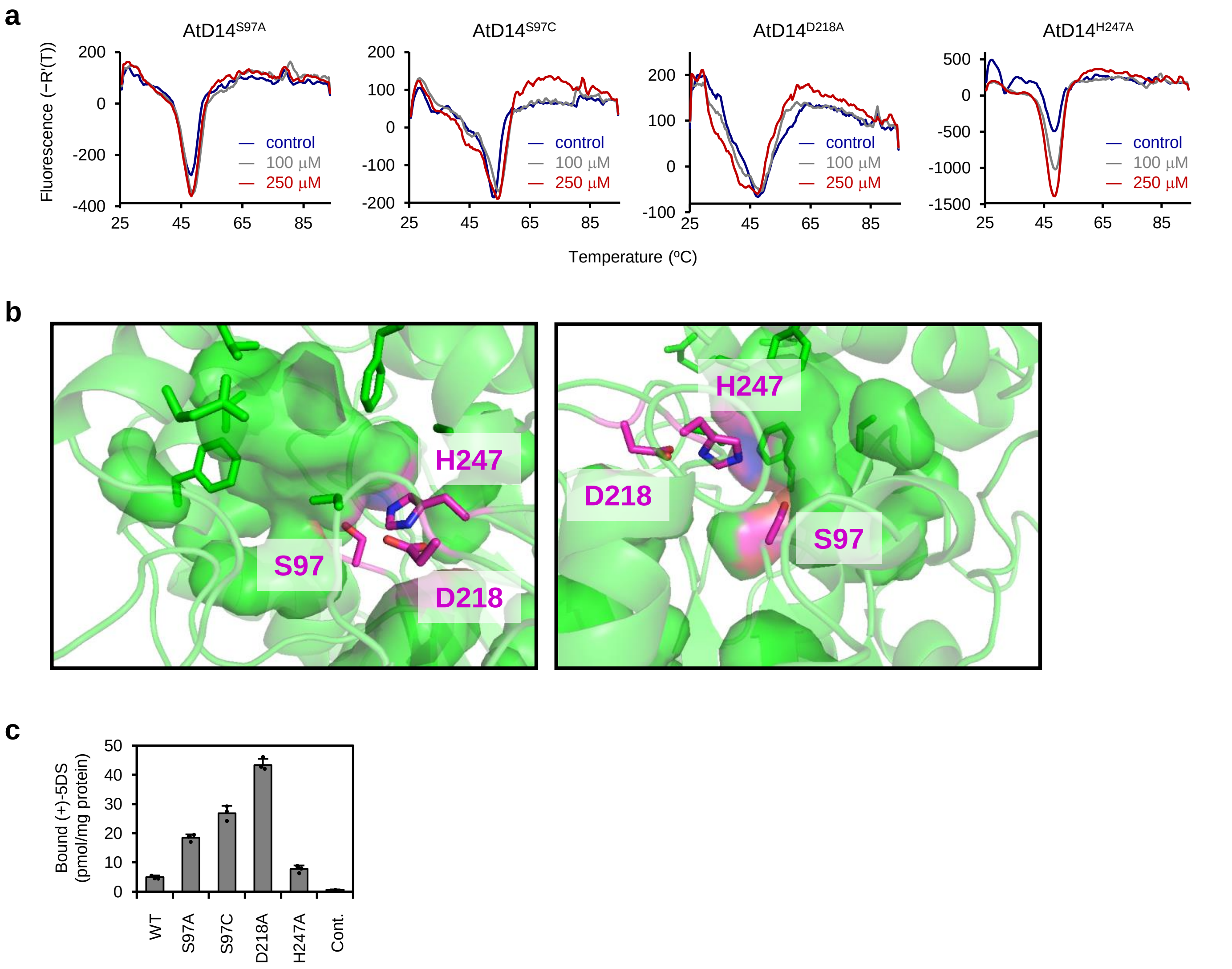

Supplementary Fig. 7. Functional analysis of catalytic triad mutants of AtD14. a, Melting temperature curves of each catalytic triad mutant of AtD14 in the presence of 5DS. **b**, Ligand binding pocket structure of the AtD14 protein. The cavity surface is shown as green sphere, and the catalytic triad amino acids are shown by magenta color. **c**, Direct binding analysis of each catalytic triad mutant of AtD14 with 5DS. Data are the means ±SD (n=3). The control (Cont.) is for MBP protein only. Source data are provided as a Source Data file.

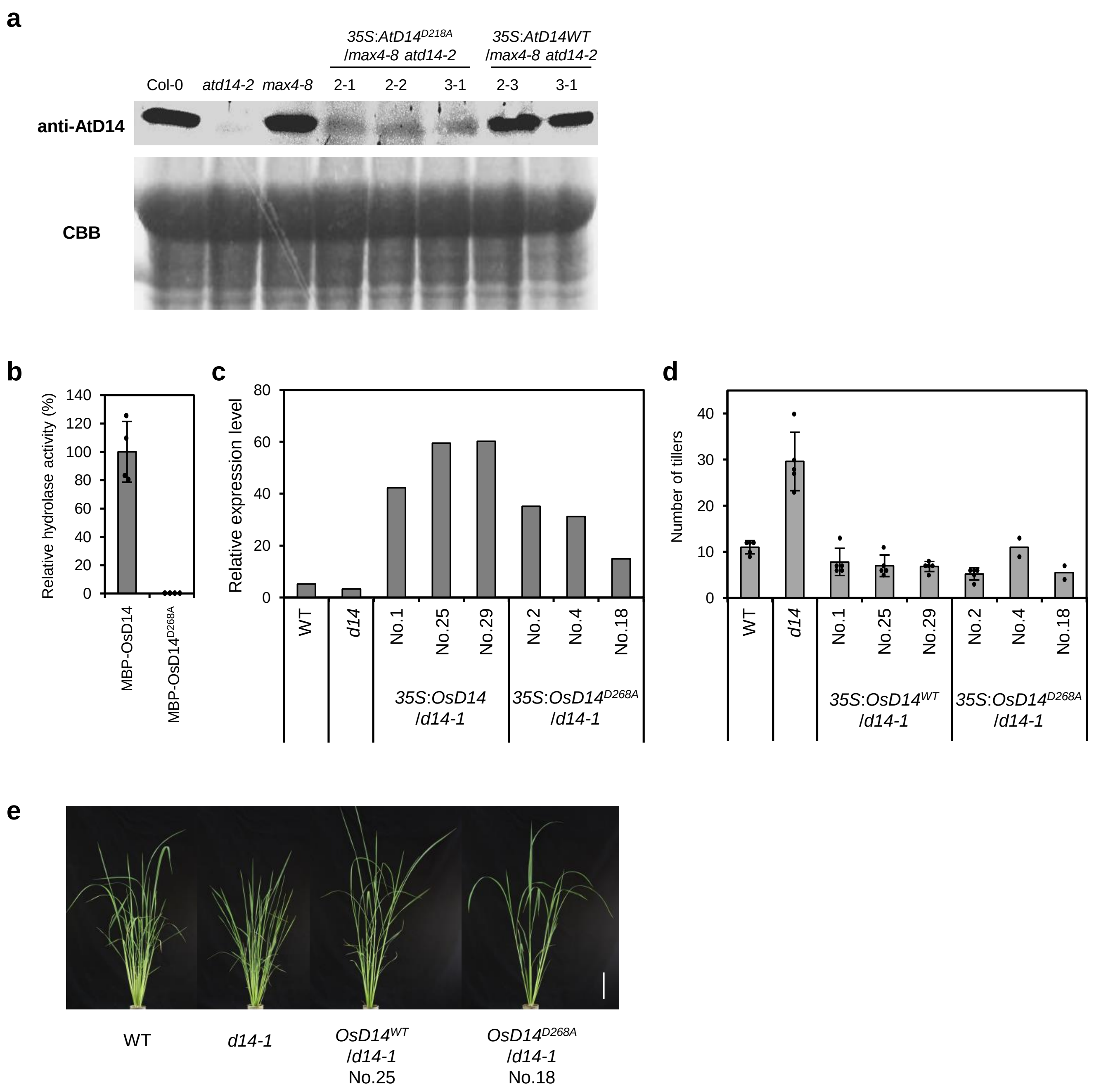

Supplementary Fig. 8. Functional analysis of the catalytic triad Asp substituted mutant of AtD14/OsD14. a, Western blotting analysis of AtD14 protein expression levels in transgenic plants expressing AtD14^{D218A} in the Arabidopsis *atd1-24 max4-8* double knockout mutant background as detected by the AtD14 antibody. **b**, Hydrolase activity of OsD14^{D268A} for 5DS at 1 μM. Data are the means ±SD (n=3). **c**, QRT-PCR analysis of *OsD14* expression levels in transgenic plants expressing *OsD14^{WT}* and *OsD14^{D268A}*, respectively, in the *d14-1* mutant background. **d**, Tiller number of 42 days old transgenic plants expressing OsD14^{WT} and OsD14^{D268A}, respectively. Data are the means ±SD (n=3-5). As for the No.4 and No. 18 of OsD14^{D268A} expressing plants, the data are average of two repeat. **e**, The pictures of 42 days old transgenic plants expressing OsD14^{WT} and OsD14^{D268A}, respectively. Scale bars = 10 cm. Source data are provided as a Source Data file. Uncropped blots can be found in the Source Data file.

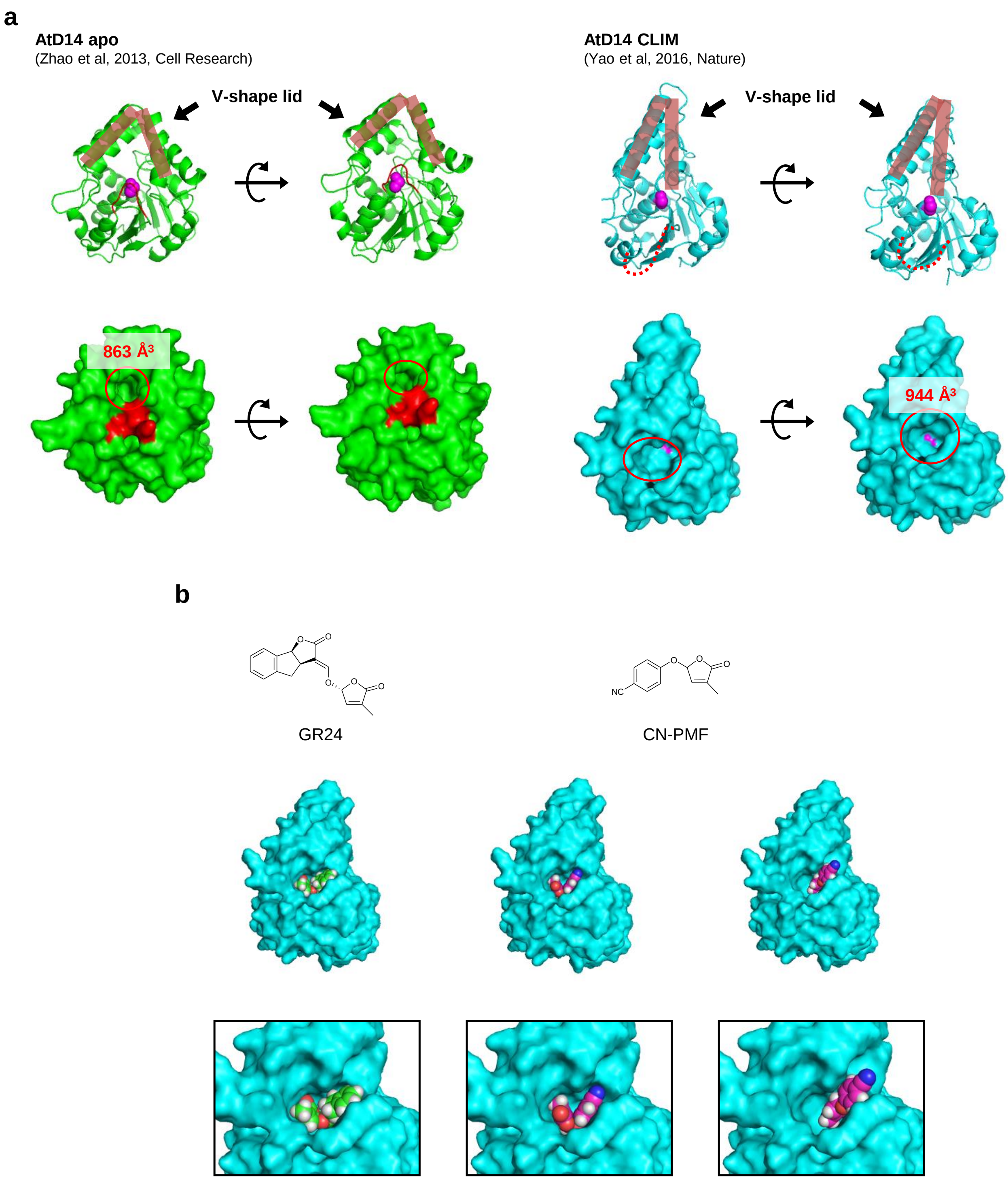

Supplementary Fig. 9. The published D14 structures. a, Structural comparison of apo-AtD14 with AtD14 in the signaling complex (AtD14 CLIM). Left; The AtD14 apo structure reported from Zhao et al (PDB code; 4IH4). The Asp loop structure is shown by the red lines. Right; The AtD14 structure in the GR24-induced complex with D3-ASK1 (PDB code; 5HZG). The estimated Asp loop structure is shown by the dashed red lines. The entrance of cavities are indicated by red circles. The amino acid indicated as magenta is the active site Ser. Volume of the cavities were calculated using CASTp program server (http://sts.bioe.uic.edu/castp/index.php). **b**, Docking structures of the conformationally altered AtD14 with SL-related compounds. A whole docking structure (upper panels), and the magnified structure around the cavity (lower panels) are shown. SWISS DOCK (http://www.swissdock.ch//) was used to obtain the binding model. A model with the lowest deltaG is shown for GR24. As for CN-PMF, two models which were similar to the GR24 biding model are shown.

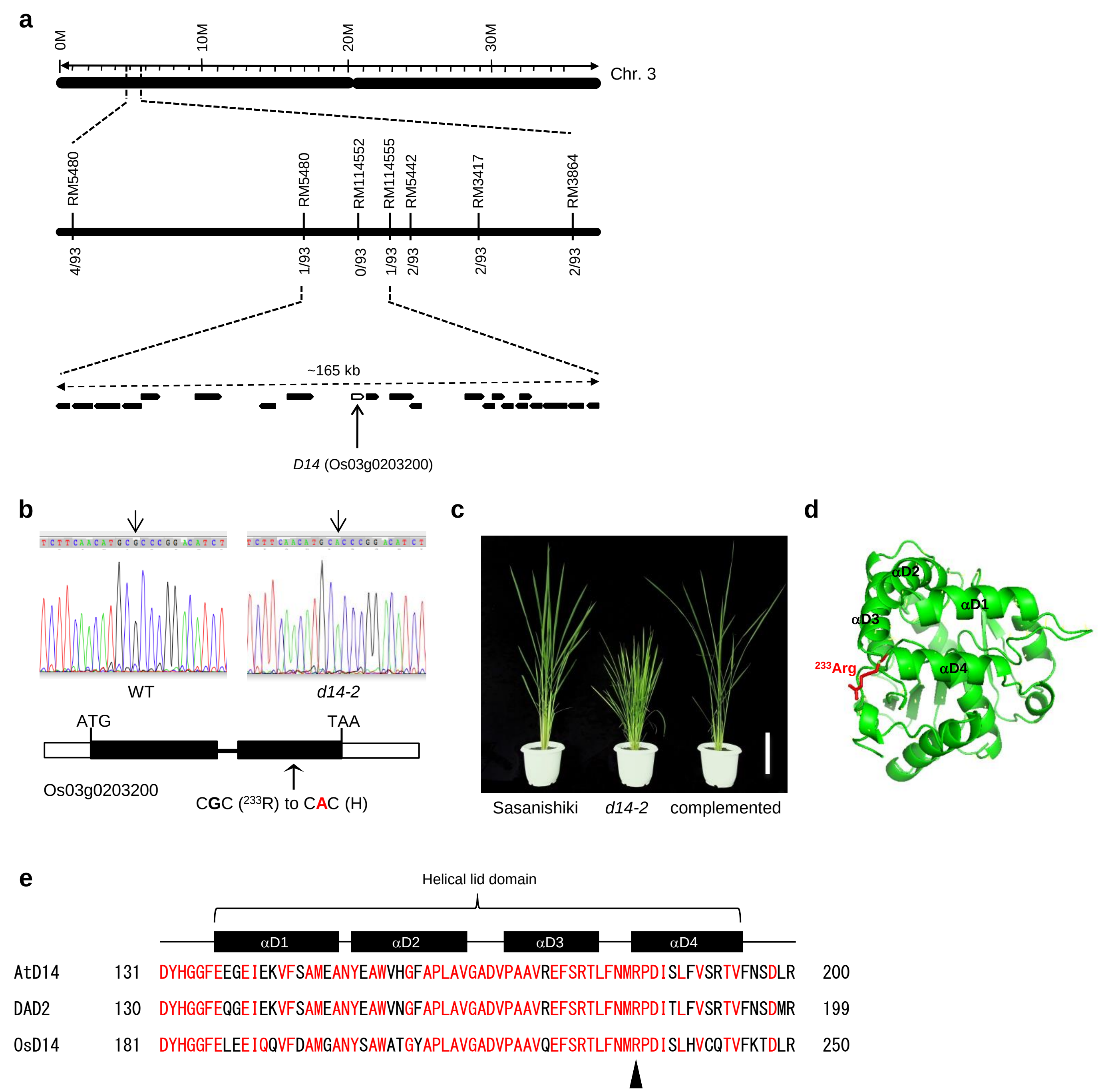

Supplementary Fig. 10. Characterization of a rice new *d14* mutant allele, *d14-2*, in Sasanishiki background. a, Fine mapping of the *OsD14* locus. Location of the mutation locus was narrowed down to a 165 kb region between RM5480 and RM114555 on chromosome 3. **b**, Upper panel; The comparison of nucleotide sequences of *OsD14* of WT (Sasanishiki) and *d14-2*, respectively. The black arrow indicates the position of the nucleotide substitution. Lower panel; Gene structure of *OsD14* (Os03g0203200). The black arrow indicates the position of the nucleotide substitution. **c**, Genetic complementation test of the *d14-2* mutant by introduction of *OsD14* gene. Scale bars = 20 cm. **d**, The reported crystal structure of OsD14 (PDB code; 3W04) showing the mutation site in the *d14-2* mutant. The red residue denotes the position of mutation site (233Arg) in the *d14-2* mutant. **e**, The amino acid sequence alignment of the helical lid domains of D14 family proteins (AtD14, DAD2, and OsD14). The red characters indicate the conserved amino acids, and a black arrow denotes the position of Arg which is substituted by His in the *d14-2* mutant.

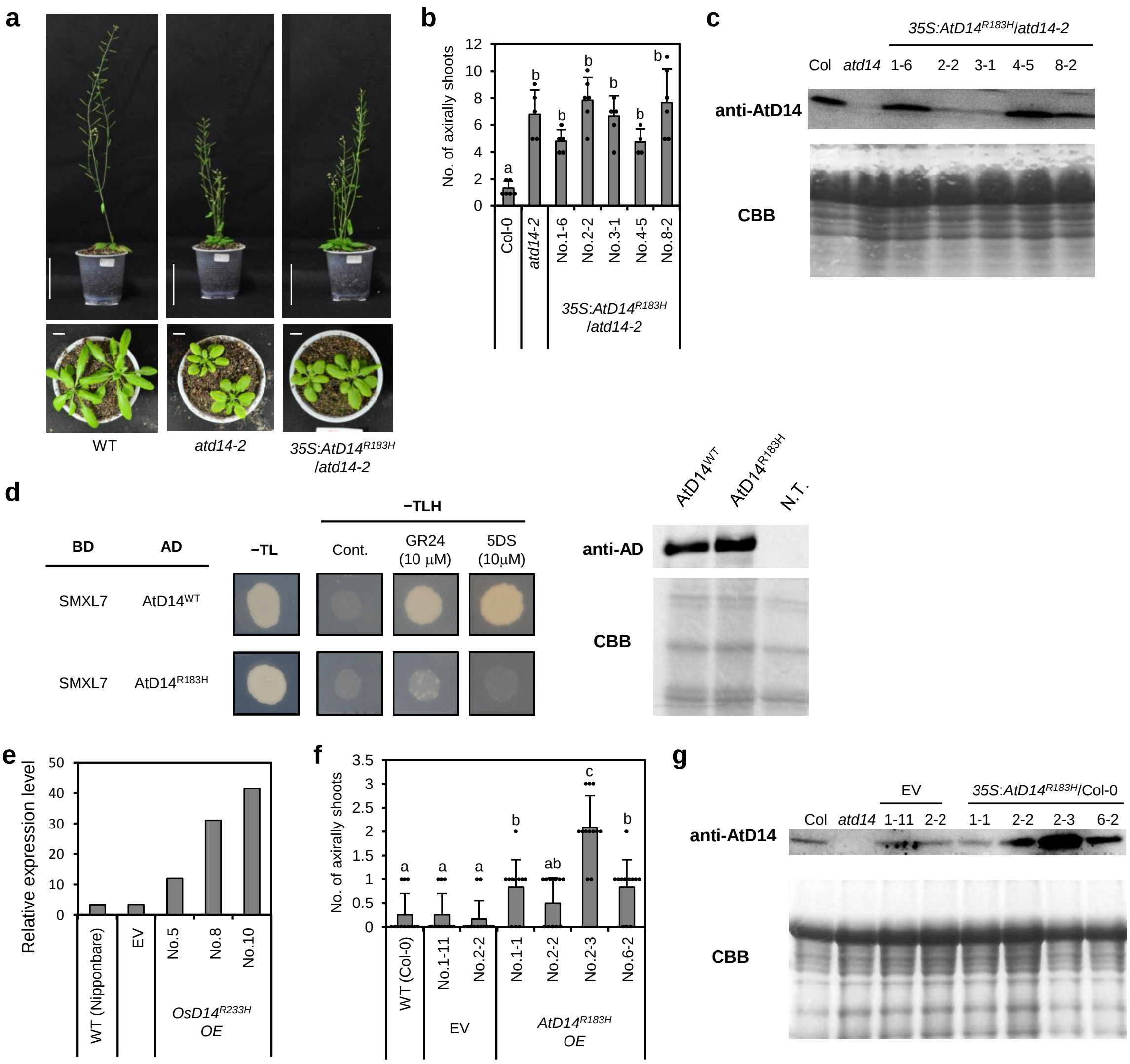

Supplementary Fig. 11. Functional analysis of OsD14^{R233H}/AtD14^{R183H} mutant protein characterized from the rice *d14-2* new mutant allele. Scale bars = 5 cm (upper panel), 1 cm (lower panel). **a**, Phenotypes of Arabidopsis transgenic plants expressing AtD14^{R183H}. Phenotypes of 50 days old mature plants (upper), and leaf morphology phenotypes of 25 days old plants (lower). **b**, No. of axillary shoots (over 5 mm) of Arabidopsis transgenic plants expressing AtD14^{R183H}. Data are the means ±SD (n=4-6). **c**, Western blot analysis of AtD14 protein expression levels in transgenic plants expressing AtD14^{R183H} in the Arabidopsis *atd14* mutant background as was detected by the AtD14 antibody. **d**, Y2H analysis of the interaction between SMXL7 and AtD14^{R183H}. Yeast transformants were spotted onto the control medium (SD −Trp/ − Leu (−TL)) and selective medium (SD −Trp/ − Leu/ − His/ (−TLH) in the absence or presence of SLs (10 μM MeCLA, or 10 μM (+)-5DS). The right panel shows the result of western blotting analysis. The expressed proteins were detected by the anti-AD antibody. N.T. indicates the nontransformed cells. **e**, QRT-PCR analysis of *OsD14* expression levels in transgenic plants expressing OsD14^{R233H} in the WT Nipponbare background under the control of 35S CaMV promoter. EV indicates the empty vector expressing plants. **f**, No. of axillary shoots (over 5 mm) of 45 days old Arabidopsis expressing AtD14^{R183H}. EV means the empty vector expressing plants. Data are the means ±SD (n=12, Different letters indicate significant differences at $P < 0.05$ with Tukey-kramer multiple comparison test.). **g**, Western blot analysis of AtD14 protein expression levels in transgenic plants expressing AtD14^{R183H} in WT Col-0 background as was detected by the AtD14 antibody. EV indicates the empty vector expressing plants. Source data are provided as a Source Data file. Uncropped blots can be found in the Source Data file.

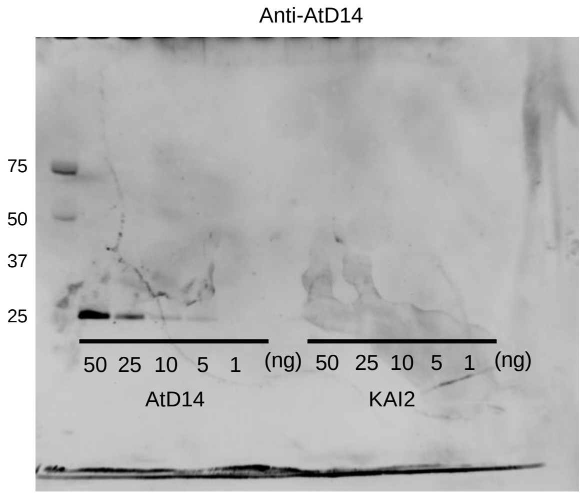

Supplementary Fig. 12. Evaluation of the AtD14 antibody using recombinant AtD14 and KAI2.

Supplementary Table 1. The nucleotide sequences of primers used in this study.

Objective	Primer name	Sequence (5'→3')
Protein expression	AtD14-F-blunt	ATGAGTCAACACAACATCTTAGAAG
	AtD14-R-EcoRI	TTTTGAATTCTCACCGAGGAAGAGC
	AtD14-S97A-F	CGTTGGTCACGCTGTTTCCG
	AtD14-S97A-R	TAAGCACAGTTTTGGATTCC
	AtD14-S97C-F	CGTTGGTCACTGTGTTTCCG
	AtD14-H248A-F	CTGAAGGAGCTTTGCCGCAG
	AtD14-H248A-R	TTTTAAGAGTCTCCACAGTAG
	AtD14-D218A-F	GGCGAAGGCTGTTTCTGTGC
	AtD14-D218A-R	GTCTGAATCACACACGTCGG
	AtD14-R183H-F	GTTTAATATGCATCCGGATATATC
	AtD14-R183H-R	AAAGTCCGGCTAAATTCTC
	OsD14-F-blunt	GCGCCGAGCGGGGCGAA
	OsD14-R-EcoR	TTTTGAATTCTTAGTACCGGGCGAG
	OsD14-D268A-F	CCGCGCCGTCTCCGTCCCGG
	OsD14-D268A-R	GTGGTCTGGACGACGACGCAGGGGG
	OsD14-R233H-F	TTCAACATGCACCCGGACATCTCCC
	OsD14-R233H-R	GAGGGTGCGGCTGAACTCCTGC
Transgenic plants generation	AtD14-F-cacc	CACCATGAGTCAACACAACA
	AtD14-R-blunt	TCACCGAGGAAGAGCTCG
	OsD14-F-cacc	CACCATGGCGCCGAGCGGGGCGAAG
	OsD14-R-blunt	TTAGTACCGGGCGAGAGCGCGG
	OsD14-genome-F	CACCGGAAGATCGCCTCATTAATCTGTT
	OsD14-genome-R	TTTTGAGATGGCAGGTGTAGGTAA
Genotyping	pGWB2-F	TATCAACAAGTTTGTACAAAAAAGC
	pGWB2-R	TATCAACCACTTTGTACAAGAAAGC
QRT-PCR	OsD14-qRT-F	CTACGTCGACGACCTGCTC
	OsD14-qRT-R	CTTGGCGAAGAGGTCAGG
	Ubiquitin-geno-F	AGAAGGAGTCCACCCTCCACC
	Ubiquitin-geno-R	GCATCCAGCACAGTAAAACACG
Y3H	ASK1-F-cacc	CACCATGTCTGCGAAGAAGATTGTG
	ASK1-R-blunt	TCATTCAAAAGCCCATTGG
	TRP1-Pro-F-NdeI	TTTTCATATGGTGCACCATAGATCAACGAC
	TRP1-R-NcoI	AAAACCATGGCTCCTTACGCATCTGTGC
	MAX2-F-BamHI	AAAAGGATCCATGGCTTCCACTACTCTCTC
	MAX2-R-PstI	TTTTCTGCAGTCAGTCAATGATGTTGCG

Supplementary Table 2. Kinetics parameters for the AtD14 protein with or without MBP.

	K_m (μM)	V_{max} (nmol/min/mg protein)	K_{cat} (1/min)
AtD14	4.9	4.0	0.12
MBP-AtD14	2.1	2.3	0.16

Supplementary Table 3. LC-MS/MS analytical conditions for SLs and those hydrolyzed products.

MS conditions

	MW	Parent ion (*m*/*z*)	Declustering potential	Collision energy (V)	column	LC condition
5DS	330	331	40	17	Acquity UPLC BEH-C18, ϕ2.1 × 50 mm, 1.7 μm	A, B
GR24	298	299	40	14	Acquity UPLC phenyl, ϕ2.1 × 50 mm, 1.7 μm	A, D
ABC-FTL (5DS type)	234	235	40	14	Acquity UPLC phenyl, ϕ2.1 × 50 mm, 1.7 μm	A
d_1 ABC-FTL (5DS type)	235	236	40	14	Acquity UPLC phenyl, ϕ2.1 × 50 mm, 1.7 μm	A
ABC-FTL (GR24 type)	202	203	40	14	Acquity UPLC phenyl, ϕ2.1 × 50 mm, 1.7 μm	A
d_1 ABC-FTL (GR24 type)	203	204	40	14	Acquity UPLC phenyl, ϕ2.1 × 50 mm, 1.7 μm	A
ABC-FTL (orobanchol type)	250	251	40	14	Acquity UPLC phenyl, ϕ2.1 × 50 mm, 1.7 μm	A
d_1 ABC-FTL (orobanchol type)	251	252	40	14	Acquity UPLC phenyl, ϕ2.1 × 50 mm, 1.7 μm	A
ABC-FTL (GR7 type)	152	153	40	14	Acquity UPLC phenyl, ϕ2.1 × 50 mm, 1.7 μm	A
d_1 ABC-FTL (GR7 type)	153	154	40	14	Acquity UPLC phenyl, ϕ2.1 × 50 mm, 1.7 μm	A
HMB	114	115	40	14	Acquity UPLC phenyl, ϕ2.1 × 50 mm, 1.7 μm	C, D
GR24 (Time Course DSF)	298	299	70	14	CORTECS UPLC C18+, ϕ2.1 × 100 mm, 1.6 μm	E
HMB (Time Course DSF)	114	115	50	13	CORTECS UPLC C18+, ϕ2.1 × 100 mm, 1.6 μm	E
CN-PMF	215	216	70	15	CORTECS UPLC C18+, ϕ2.1 × 100 mm, 1.6 μm	E
4-Hydroxybenzonitrile (HBN)	119	120	70	27	CORTECS UPLC C18+, ϕ2.1 × 100 mm, 1.6 μm	E
1-Naphthalene acetic acid (NAA)	186	187	70	40	CORTECS UPLC C18+, ϕ2.1 × 100 mm, 1.6 μm	E
ABC-FTL (GR24 type) (Time Course DSF)	202	203	70	15	Acquity UPLC HSS T3, ϕ2.1 × 50 mm, 1.8 μm	F
d_1ABC-FTL (GR24 type) (Time Course DSF)	203	204	70	15	Acquity UPLC HSS T3, ϕ2.1 × 50 mm, 1.8 μm	F
AtD14 protein	-	-	80	10	Acquity UPLC BEH-C4, ϕ2.1 × 50 mm, 1.7 μm	G

LC conditions

Condition A

Time (min)	Solvent A	Solvent B
0.10	65	35
4.00	20	80
4.01	2	98
4.50	2	98

Condition B

Time (min)	Solvent A	Solvent B
0.10	70	30
1.00	60	40
6.00	30	70
6.01	2	98

Condition C

Time (min)	Solvent A	Solvent B
0.10	99	1
5.00	93	7
5.01	0	100

Condition D

Time (min)	Solvent A	Solvent B
0.50	99	1
2.00	95	5
4.50	2	98
6.00	2	98

Condition E

Time (min)	Solvent A	Solvent B
0.00	100	0
8.00	20	80
8.10	2	98
9.60	2	98

Condition F

Time (min)	Solvent A	Solvent B
0.00	90	10
6.00	30	70
6.10	2	98
7.00	2	98

Condition G

Time (min)	Solvent C	Solvent D
0.00	97	3
7.00	49	51
7.10	2	98
9.80	2	98

Solvent A = Water (0.05% AcOH)
Solvent B = Acetonitrile (0.05% AcOH)
Solvent C = Water (0.1% formic acid)
Solvent D = Acetonitrile (0.1% formic acid)
